# Crosslinking of a Peritrophic Matrix Protein Protects Gut Epithelia from Bacterial Exotoxins

**DOI:** 10.1371/journal.ppat.1005244

**Published:** 2015-10-27

**Authors:** Toshio Shibata, Kouki Maki, Jinki Hadano, Takumi Fujikawa, Kazuki Kitazaki, Takumi Koshiba, Shun-ichiro Kawabata

**Affiliations:** 1 Department of Biology, Faculty of Science, Kyushu University, Fukuoka, Japan; 2 Institute for Advanced Study, Kyushu University, Fukuoka, Japan; 3 Graduate School of Systems Life Sciences, Kyushu University, Fukuoka, Japan; Stanford University, UNITED STATES

## Abstract

Transglutaminase (TG) catalyzes protein-protein crosslinking, which has important and diverse roles in vertebrates and invertebrates. Here we demonstrate that *Drosophila* TG crosslinks drosocrystallin, a peritrophic matrix protein, to form a stable fiber structure on the gut peritrophic matrix. RNA interference (RNAi) of the *TG* gene was highly lethal in flies and induced apoptosis of gut epithelial cells after oral infection with *Pseudomonas entomophila*. Moreover, AprA, a metalloprotease secreted by *P*. *entomophila*, digested non-crosslinked drosocrystallin fibers, but not drosocrystallin fibers crosslinked by TG. *In vitro* experiments using recombinant drosocrystallin and monalysin proteins demonstrated that monalysin, a pore-forming exotoxin of *P*. *entomophila*, was adsorbed on the crosslinked drosocrystallin fibers in the presence of *P*. *entomophila* culture supernatant. In addition, gut-specific *TG*-RNAi flies had a shorter lifespan than control flies after ingesting *P*. *entomophila*, whereas the lifespan after ingesting *AprA*-knockout *P*. *entomophila* was at control levels. We conclude that drosocrystallin fibers crosslinked by TG, but not non-crosslinked drosocrystallin fibers, form an important physical barrier against exotoxins of invading pathogenic microbes.

## Author Summary

Intestinal homeostasis is ensured by a subtle balance between bacteria and host immunity. Gut epithelial barriers, such as the mucus layer in mammals and the peritrophic matrix in invertebrates, have a protective function for the host, as they are impermeable to invading intestinal microbes. Here we found that, in the fly *Drosophila melanogaster*, transglutaminase (TG), a molecular glue involved in protein-protein covalent bond formation, is essential for peritrophic matrix formation by converting the peritrophic protein drosocrystallin into a stable fiber-like structure and inhibition of pathogenic bacteria. Knockdown of the *TG* gene led to increased permeability of the peritrophic matrix and greatly increased the susceptibility to a toxic bacterial protease. TG contributes to form a stable fiber-like barrier on the peritrophic matrix and increase tolerance to pathogenic microorganisms.

## Introduction

Gut epithelia are the first line of defense against invading microorganisms. *Drosophila* has several gut defense systems, including the production of antimicrobial peptides [1–3] and reactive oxygen species [4–6], peritrophic matrix formation [7], and stem cell activation for cell renewal [8,9]. Transglutaminase (TG) is involved in the regulation of antimicrobial peptide production and peritrophic matrix formation [10]. TG catalyzes the isopeptide bond formation between lysine and glutamine residues, and has diverse physiologic roles in vertebrates and invertebrates [11,12]. *Drosophila* TG is involved in cuticular formation [13] and hemolymph coagulation, which traps invading pathogens [14,15]. The concept of hemolymph coagulation in invertebrates as a part of the early innate immune system has been extended to vertebrates [14,15]. Recently, we reported that systemic and gut-specific *TG*-knockdown flies have a shorter lifespan than control flies, concomitant with severe apoptosis of cells in the gut epithelium [10]. Moreover, we found that TG crosslinks N-terminal Relish in the immune deficiency pathway to suppress antimicrobial peptide expression, thereby enabling immune tolerance against gut microbes. Further, RNA interference (RNAi) of the *TG* gene causes peritrophic matrix defects and penetration of dextran beads from the gut lumen (endoperitrophic space) into the ectoperitrophic space [10].

The peritrophic matrix in insects is a non-cellular sieve-like structure that lines the midgut epithelium, and comprises chitin fibrils and chitin-binding proteins [16]. This matrix has a role analogous to that of the mucosal layer of the vertebrate intestine, and is thought to support digestion and provide protection against abrasive food particles and enteric pathogens [17]. The *drosocrystallin* gene, which encodes a 52-kDa glycoprotein with Ca^2+^-binding ability, was originally identified in *Drosophila* eyes, but its function was not clear [18,19]. Drosocrystallin was recently reported to have an important role in protecting against entomopathogenic bacteria such as *Pseudomonas entomophila* [7]. Drosocrystallin expression is induced upon oral infection by bacteria, and the peritrophic matrix of *drosocrystallin*-knockout flies is more permeable than that of wild-type flies, demonstrating the essential role of drosocrystallin in peritrophic matrix formation [7]. Drosocrystallin is a secreted glycoprotein containing a chitin-binding R&R motif [20,21]. Cuticular chitin-binding proteins in horseshoe crabs are substrates for TG [22], suggesting that drosocrystallin could be a potential TG substrate. Here we demonstrate that TG enhances the structural strength of the peritrophic matrix by crosslinking drosocrystallin fibers, and that the crosslinked drosocrystallin fibers, but not non-crosslinked drosocrystallin fibers, are essential for protection against exotoxins secreted by gut-invading pathogenic bacteria.

## Results and Discussion

### Drosocrystallin is crosslinked by TG in vitro and in vivo

**Fig 1:**
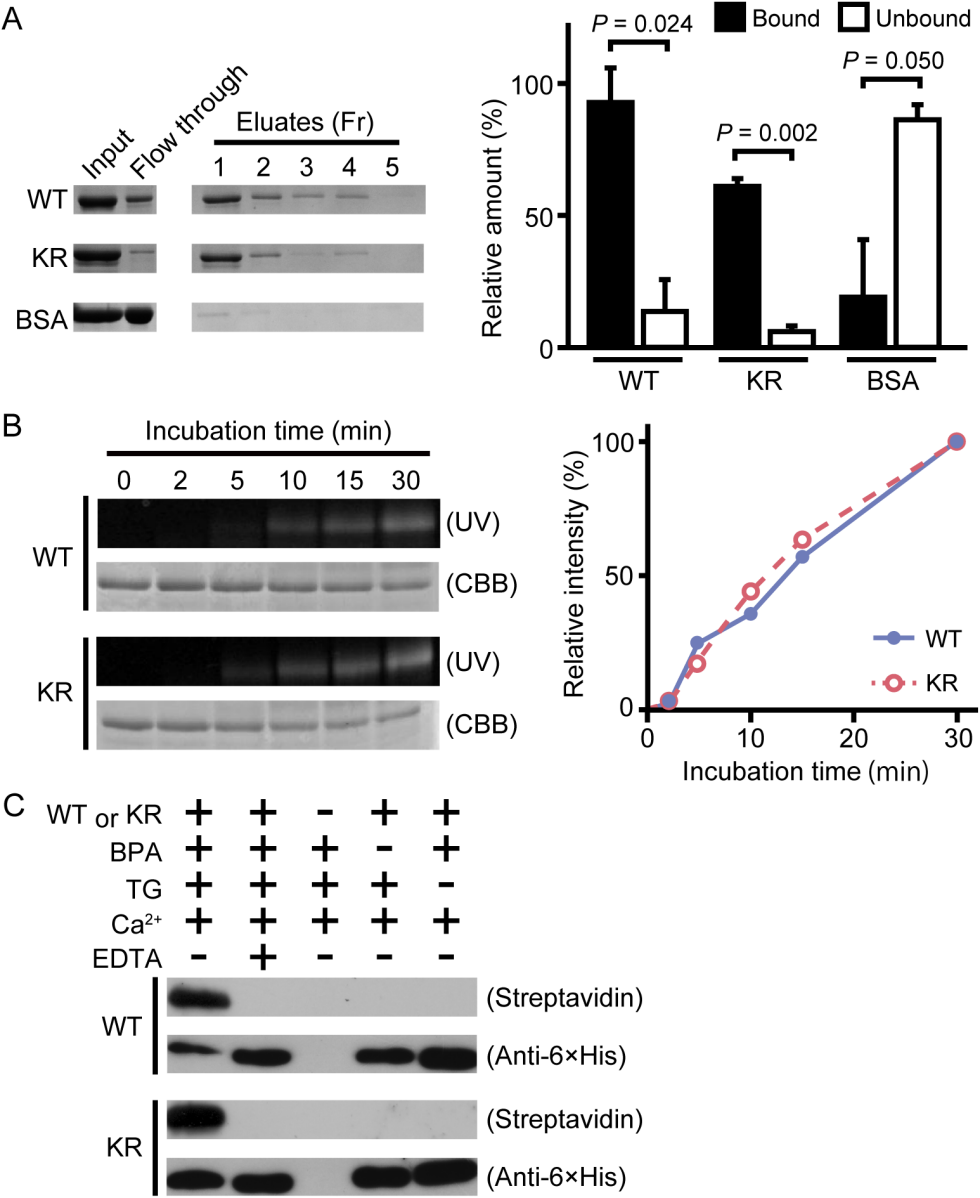
TG-dependent incorporation of monodansylcadaverine (MDC) or biotin pentylamine into drosocrystallin recombinants. (A) Wild-type drosocrystallin (WT), the KR mutant (KR), or bovine serum albumin (BSA) was incubated with chitin, and each fraction (Fr) was analyzed by SDS-PAGE in 10% slab gels (left panel). The intensity of each fraction relative to that of the input was summed as the bound fraction. Bars indicate the mean and standard deviations of experiments performed in triplicate (right panel). BSA was used as a negative control. Open bars, unbound fraction; closed bars, bound fraction. (B) Wild-type drosocrystallin (WT) or the KR mutant (KR) was incubated with MDC in the presence of TG, and analyzed by SDS-PAGE in 10% slab gels. The proteins were stained with Coomassie brilliant blue (CBB), and the MDC-incorporated protein was detected by the emission intensity of the dansyl group. Data are representative of three independent experiments (left panel). The relative emission intensity of each fraction compared to that of CBB-stained protein was calculated using ImageJ software (right panel). (C) Wild-type drosocrystallin (WT) or the KR mutant (KR) was incubated with or without biotin pentylamine (BPA) in the presence of TG, and subjected to SDS-PAGE in 10% slab gels. Incorporation of BPA was detected with horseradish peroxidase-conjugated streptavidin. Loaded recombinant proteins were detected by Western blotting with a horseradish peroxidase-conjugated anti-6× His tag antibody. Data are representative of at least three independent experiments.

**Fig 2:**
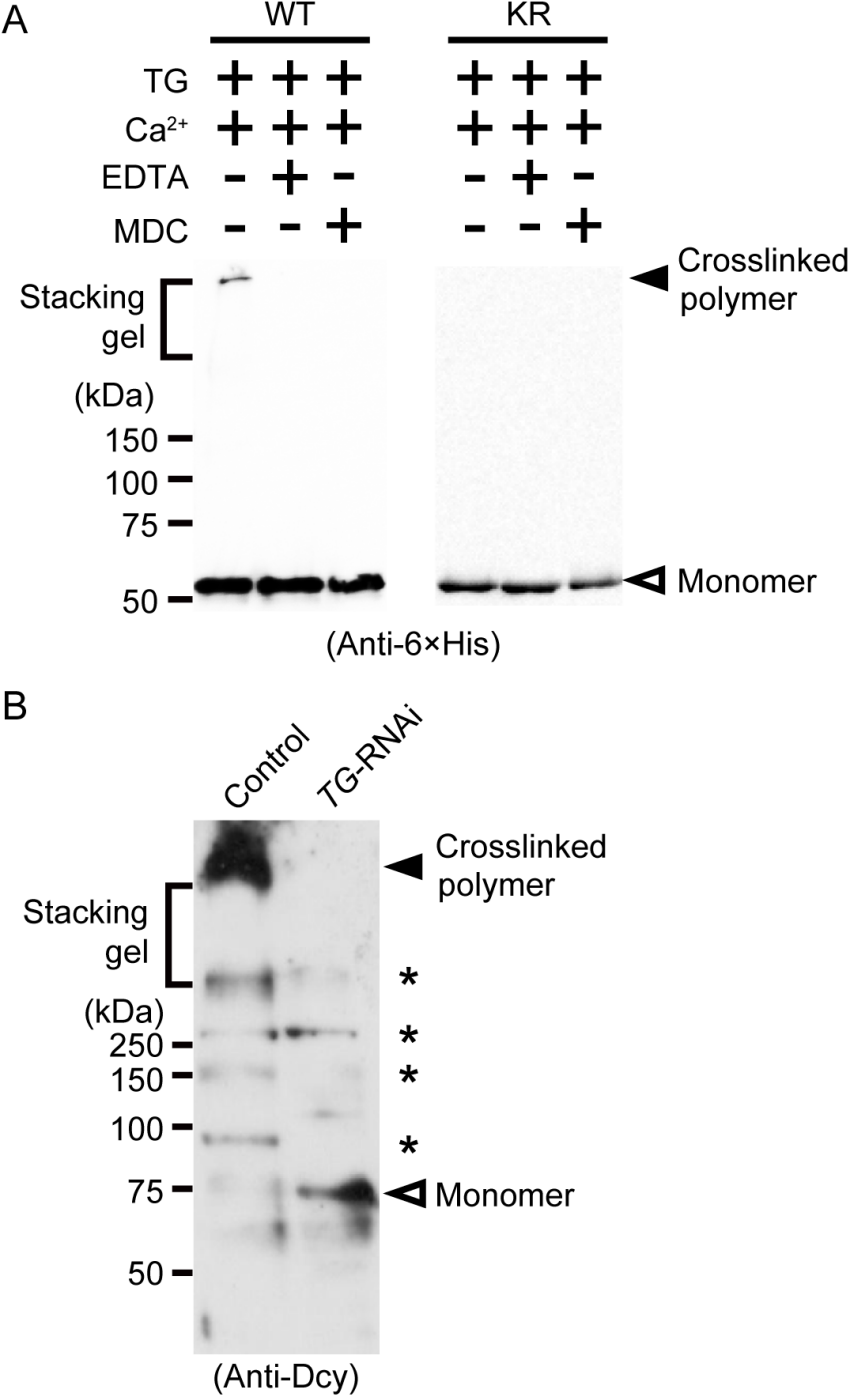
TG-dependent polymerization of drosocrystallin *in vitro* and *in vivo*. **(**A) Wild-type drosocrystallin (WT) or the KR mutant (KR) was incubated with TG, and subjected to SDS-PAGE in 10% TGX FastCast gels (Bio-Rad Laboratories). These recombinants were detected by Western blotting with a horseradish peroxidase-conjugated anti-6 × His tag antibody. Monodansylcadaverine (MDC) was used as an inhibitor of protein-protein crosslinking. Data are representative of at least three independent experiments. **(**B) Gut extracts from systemic *TG-*RNAi flies (*Da>TG IR*) and their counterparts (*Da>+*) were subjected to SDS-PAGE in 12% slab gels. Native drosocrystallin in the extracts was detected by Western blotting with an anti-drosocrystallin antibody (Anti-Dcy). Asterisks indicate unknown cross-reacted proteins. Data are representative of three independent experiments.

Wild-type drosocrystallin and a lysine-to-arginine substituted mutant (KR) were prepared in *Escherichia coli*. To examine the functional activity of these recombinants, we evaluated chitin-binding activity. Both recombinants clearly bound to chitin (Fig 1A, left and right panels). The recombinants were then incubated with TG in the presence of monodansylcadaverine (MDC) or biotin pentylamine, an amino-substrate of TG. MDC was incorporated into these recombinants in a time-dependent manner (Fig 1B, left panel). MDC was incorporated into the KR mutant at the same rate as in the wild-type recombinant (Fig 1B, right panel), because the amino-substrate is incorporated in glutamine residues. The biotin pentylamine-incorporated recombinants were also detected using streptavidin, but incorporation was inhibited in the presence of a TG inhibitor, EDTA (Fig 1C). In wild-type drosocrystallin, a protein band was observed on the stacking gel, but not in the KR mutant in the absence of MDC and EDTA (Fig 2A), indicating that drosocrystallin is covalently crosslinked by TG to form homopolymers. To clarify the crosslinking profile of drosocrystallin *in vivo*, we generated systemic *TG*-knockdown flies (*Da>TG IR*). Western blotting using anti-drosocrystallin antibody revealed a protein band on the stacking gel in the gut of control flies (*Da>+*), but not *TG*-knockdown flies (Fig 2B), indicating that drosocrystallin was covalently crosslinked by TG *in vivo*. In sodium dodecyl sulfate-polyacrylamide gel electrophoresis (SDS-PAGE), the non-glycosylated wild-type recombinant expressed in *E*. *coli* and intact drosocrystallin in the gut had apparent molecular masses of 52 kDa and 75 kDa, respectively (Fig 2A and 2B).

### Crosslinked drosocrystallin, but not non-crosslinked drosocrystallin, is resistant to proteases secreted by pathogenic bacteria

**Fig 3:**
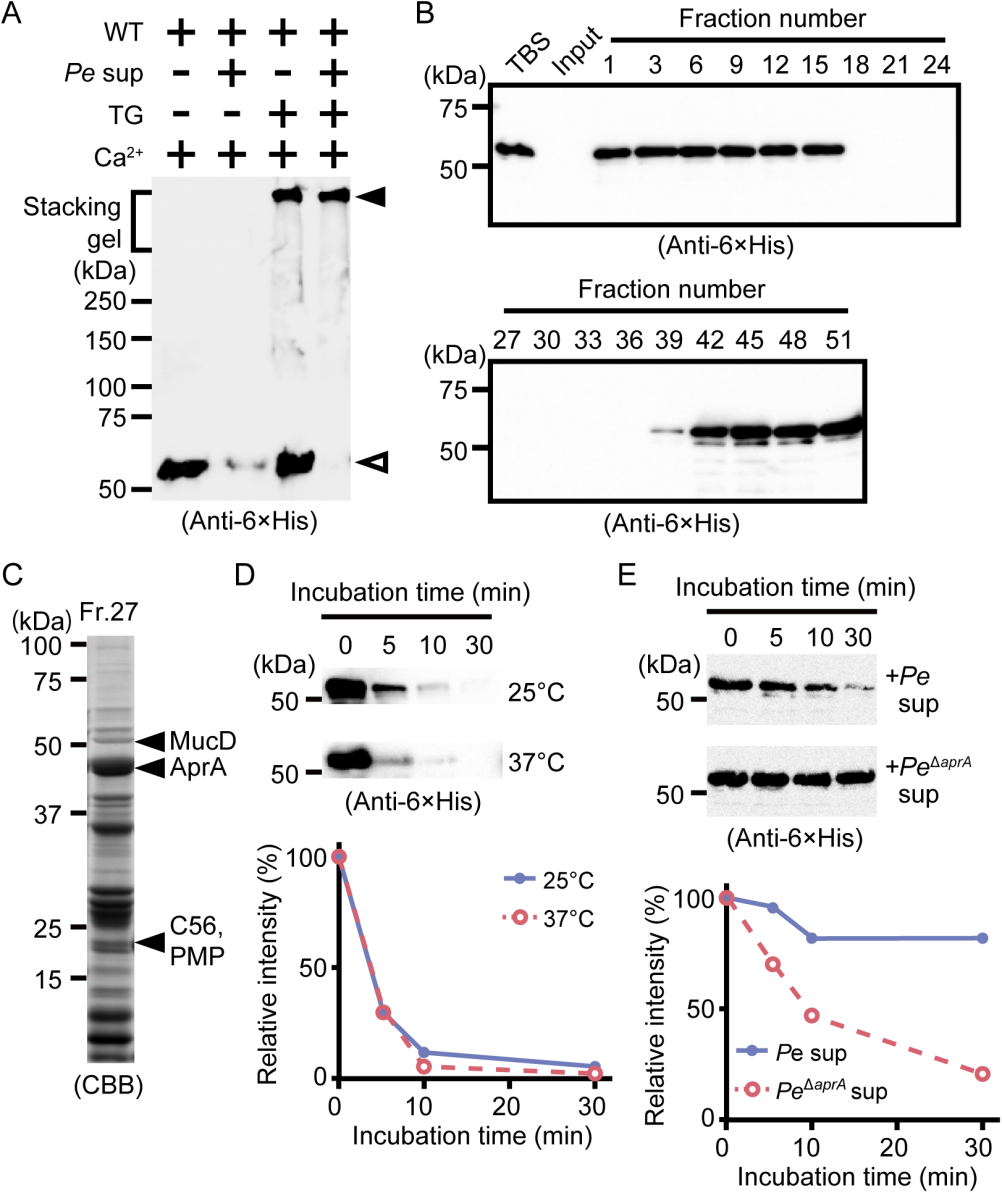
Polymerized drosocrystallin protects against AprA. (A) Wild-type drosocrystallin (WT) was incubated at 37°C for 30 min with or without TG, and then the culture supernatant from *P*. *entomophila* (*Pe*) was added, and the mixture was subjected to SDS-PAGE in 10% TGX FastCast gels. Open arrowhead, the monomeric recombinant; closed arrowhead, the crosslinked recombinant. Data are representative of at least three independent experiments. (B) Wild-type drosocrystallin was subjected to SDS-PAGE in 10% slab gels and detected by Western blotting after incubating with each fraction obtained by gel filtration of the culture supernatant from *P*. *entomophila*. (C) Fraction No. 27 from the gel filtration was subjected to SDS-PAGE in 15% slab gels, and proteases in this fraction were identified by liquid chromatography tandem mass spectroscopy analysis. (D) Wild-type drosocrystallin was incubated with purified AprA at 25°C or 37°C and analyzed by SDS-PAGE in 10% slab gels, and detected by Western blotting using anti-6 × His tag antibody (upper panel). Western blotting data are representative of four independent experiments. The relative intensity of each band compared to that of the untreated protein (0 min) was calculated using ImageJ software (lower panel). (E) Wild-type drosocrystallin was incubated with the culture supernatant from *P*. *entomophila* (*Pe*) or the *AprA*-knockout strain (*Pe* ^*ΔaprA*^), analyzed by SDS-PAGE in 10% slab gels, and detected by Western blotting using anti-6 × His tag antibody. Western blotting data are representative of three independent experiments (upper panel). The relative intensity of each band compared to that of the untreated protein (0 min) was calculated using ImageJ software (lower panel).

Drosocrystallin is important for host defense against bacterial protease AprA secreted by *P*. *entomophila* [7]. Protease AprA is a metalloprotease important for local infection [2,23,24]. Wild-type drosocrystallin was degraded by adding the culture supernatant from *P*. *entomophila* in the absence of TG, but recombinant drosocrystallin crosslinked by TG was not degraded (Fig 3A). In *Drosophila*, TG-mediated crosslinking of Fondue and hexamerin is important for trapping invading microbes in the hemocoel [14,25]. In the case of *Trichoplusia ni*, the chitin-binding proteins (CBP1 and CBP2) and the insect intestinal mucin bind to chitin fibers on the peritrophic matrix, which protect the insect from food-derived digestive proteases [26–28]. These previous findings together with our results indicate that crosslinked drosocrystallin on the peritrophic matrix could act as a protective physical barrier against *P*. *entomophila* exotoxins.

To identify the pathogenic protease(s) involved in the degradation of non-crosslinked drosocrystallin, the culture supernatant from *P*. *entomophila* was fractionated by gel filtration, and wild-type drosocrystallin was incubated with each fraction. The non-crosslinked recombinant was not detected in the fractions (Nos. 18–36) by Western blotting, possibly due to proteolytic digestion (Fig 3B). Metalloprotease AprA [2,23,24] and three proteases with unknown function, including MucD, C56, and PMP, were identified from one fraction (No. 27) by mass spectrometry, and AprA was the most abundant protease in this fraction (Fig 3C). Therefore, we purified AprA from a fraction (No. 26), as described in the Materials and Methods. Wild-type drosocrystallin was completely degraded by the purified AprA (Fig 3D). To confirm the involvement of AprA in the digestion of drosocrystallin, culture supernatant without AprA was prepared using *AprA*-knockout *P*. *entomophila*. The resulting supernatant did not significantly degrade wild-type drosocrystallin (Fig 3E, upper and lower panels), clearly indicating that AprA is a key protease for the degradation of non-crosslinked drosocrystallin.

### Drosocrystallin fibers stabilized by TG-catalyzed crosslinking are resistant to bacterial toxins

**Fig 4:**
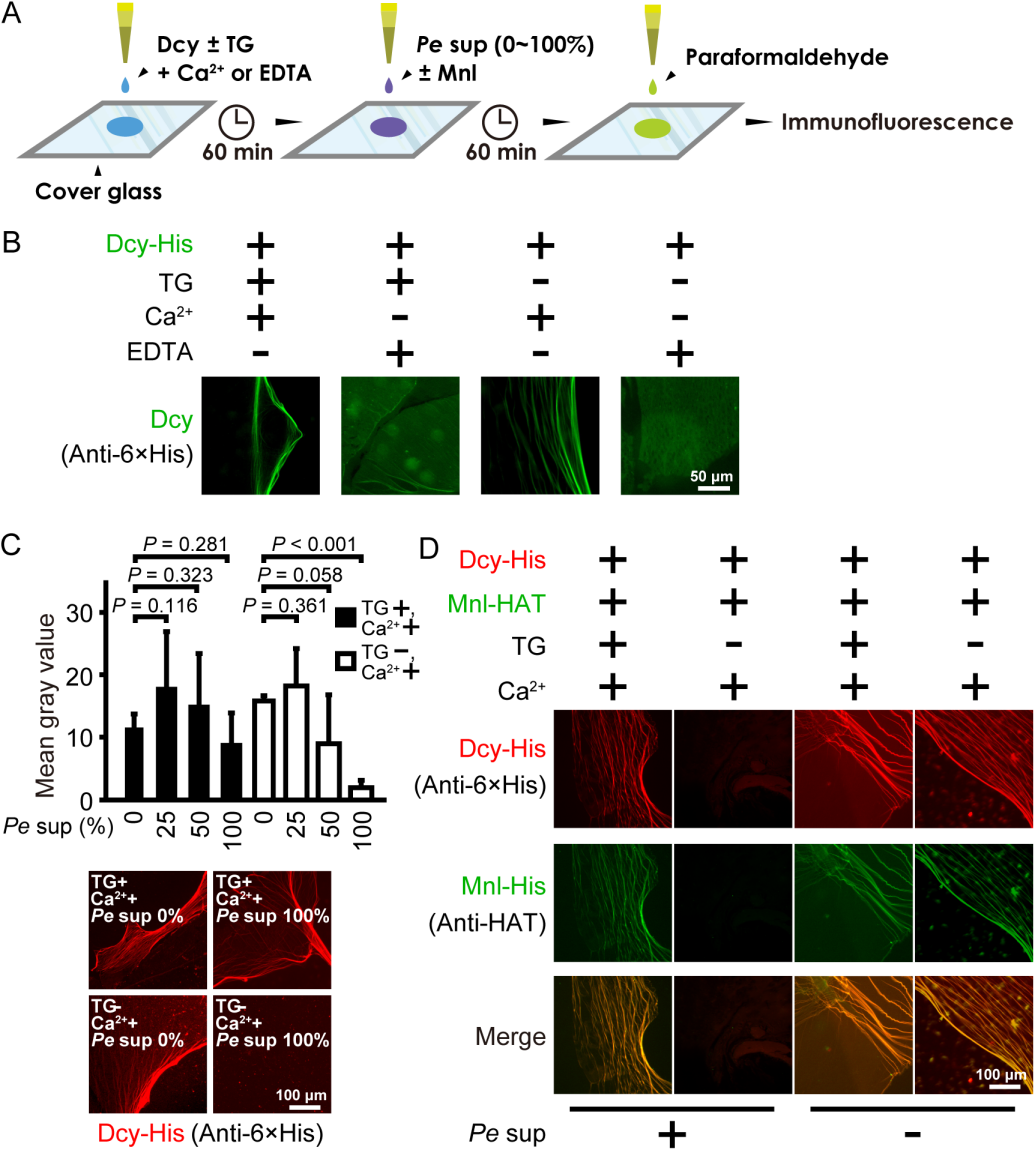
Covalently crosslinked drosocrystallin fibers are resistant to proteolytic digestion and trap a pore-forming exotoxin, monalysin. (A) A schematic of the assay for the fiber formation of drosocrystallin. Briefly, wild-type drosocrystallin (Dcy) was incubated on cover glass in the presence or absence of TG and Ca^2+^ or EDTA for 1 h. The culture supernatant from *P*. *entomophila* (*Pe* sup) containing protease AprA was added on the cover glass with or without monalysin (Mnl). After incubation, proteins on the cover glass were fixed with paraformaldehyde and detected by immunofluorescence. (B) Wild-type drosocrystallin was incubated on coverslip glass and observed by immunofluorescence microscopy. Wild-type drosocrystallin was detected using an anti-His tag antibody and CF488-conjugated secondary antibody (green). Representative data from at least five experiments are shown. (C) Wild-type drosocrystallin was incubated with or without TG on coverslip glass in the presence of Ca^2+^, and then 2-fold serial dilutions of the culture supernatant from *P*. *entomophila* (*Pe* sup) were added. The concentration of the culture supernatant is indicated. Bars indicate the mean and standard deviations of the mean gray values from four independent experiments (upper panel). Lower panels show representative data of drosocrystallin fibers formed in the presence or absence of TG and *Pe* sup. Wild-type drosocrystallin was detected by anti-His tag antibody and CF568-conjugated secondary antibody (red). (D) Wild-type drosocrystallin was incubated with or without TG on coverslip glass in the presence of Ca^2+^, and then wild-type monalysin was added in the presence (*Pe* sup +) or absence (*Pe* sup −) of the culture supernatant from *P*. *entomophila*. Wild-type drosocrystallin was detected by anti-His tag antibody and CF568-conjugated secondary antibody (red), and wild-type monalysin was detected by anti-histidine affinity tag (HAT) antibody and CF488-conjugated secondary antibody (green). One representative experiment from at least five independent experiments is shown.

TG-catalyzed fibers or a mesh formation of several proteins, such as proxin and stablin in horseshoe crabs, is important for wound healing and bacterial entrapment [29,30]. To determine whether drosocrystallin forms fibers or a mesh by protein-protein crosslinking of TG activity, wild-type drosocrystallin was incubated on cover glass under several conditions and observed by immunofluorescence microscopy (Fig 4A). Interestingly, fiber-like structures were observed in the absence of TG, but the fibers were not detected in the presence of EDTA, indicating that Ca^2+^ induces non-covalent self-association of drosocrystallin (Fig 4B). This finding is consistent with a previous finding that drosocrystallin exhibits Ca^2+^-binding ability [19], and suggests that Ca^2+^ is required not only for TG activation, but also for non-covalent fiber formation of drosocrystallin. To clarify the importance of covalent crosslinking of drosocrystallin mediated by TG, the effect of culture supernatant including protease AprA from *P*. *entomophila* on the stability of drosocrystallin fibers was observed with or without active TG. In the absence of active TG, the fiber structure of wild-type drosocrystallin gradually collapsed in proportion to the amount of the culture supernatant from *P*. *entomophila* and the fluorescence intensity of the fibers decreased further (Fig 4C). On the other hand, in the presence of active TG, the fiber structure and fluorescence intensity of drosocrystallin were not affected by culture supernatant of *P*. *entomophila*. These findings suggest that TG-mediated covalent crosslinking of drosocrystallin is required for the protection against proteolytic digestion. *P*. *entomophila* secretes another exotoxin, monalysin, that acts as a pore-forming toxin against cell membranes, causing host cell death [24]. To examine whether the crosslinked fibers of drosocrystallin protect against penetration of monalysin into the peritrophic matrix, crosslinked or non-crosslinked fibers of wild-type drosocrystallin were mixed with wild-type monalysin in the presence or absence of the culture supernatant, and both recombinants were observed by immunofluorescence microscopy. Wild-type monalysin colocalized with the crosslinked fibers of wild-type drosocrystallin (Fig 4D, left panels of *Pe* sup +). In contrast, the non-crosslinked fibers in the absence of TG were degraded by proteases in the culture supernatant and wild-type monalysin did not colocalize with wild-type drosocrystallin (Fig 4D, right panels of *Pe* sup +). In the absence of the culture supernatant, however, wild-type monalysin colocalized with both the TG-dependent crosslinked fibers and the Ca^2+^-induced non-covalent associated fibers of wild-type drosocrystallin (Fig 4D, left and right panels of *Pe* sup −). These findings indicate that the non-covalent fiber formation of drosocrystallin leads to co-localization of monalysin and that the protease-resistant drosocrystallin fibers crosslinked by TG, but not non-crosslinked drosocrystallin fibers, trap monalysin released from *P*. *entomophila* in the presence of proteases such as metalloprotease AprA.

### TG is important for protection against protease AprA

**Fig 5:**
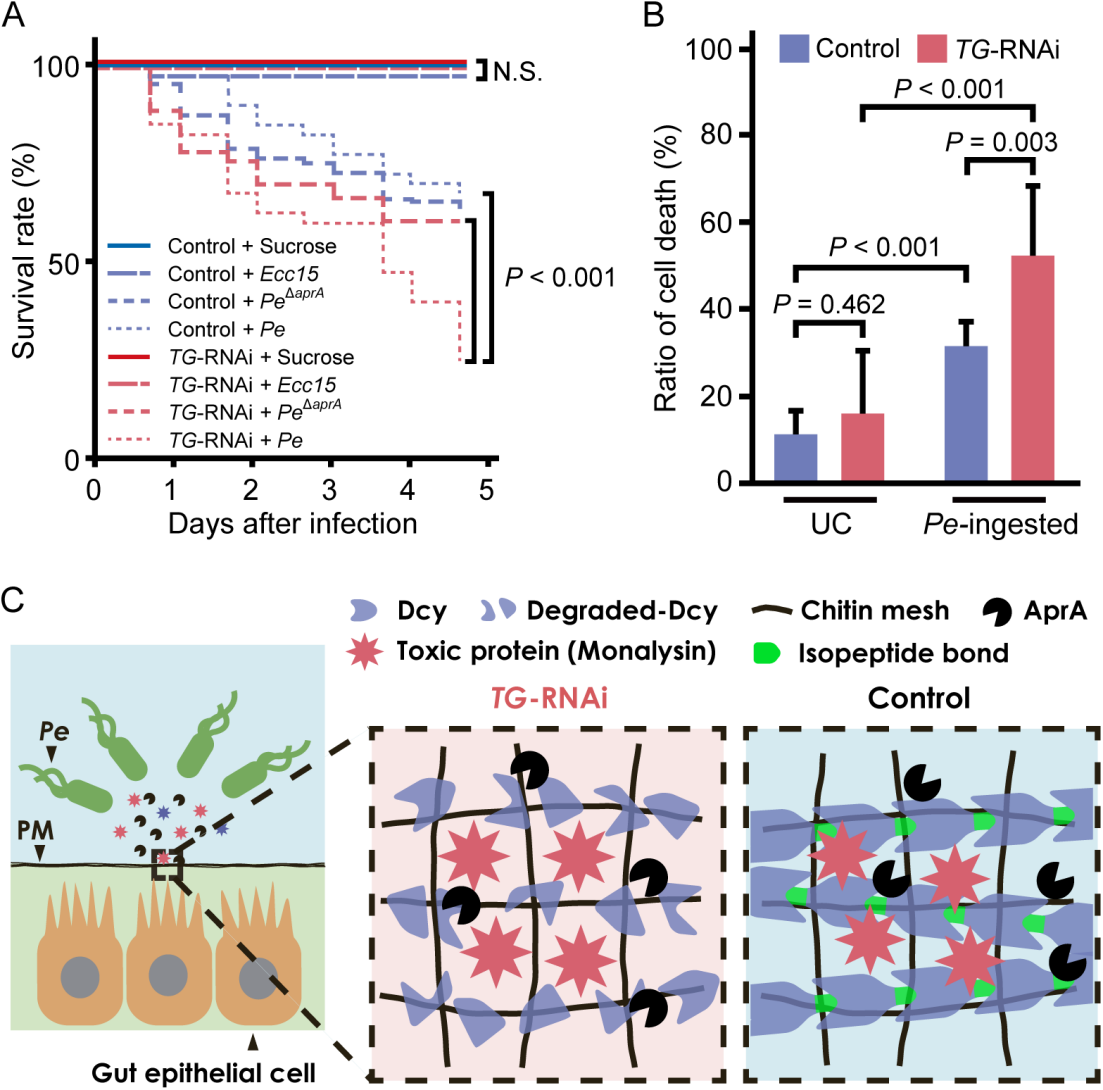
TG-dependent protection against *P*. *entomophila* infection in the gut. (A) Survival analysis of gut-specific *TG-*RNAi flies (*NP1>TG IR*) and their counterparts (*NP1>+*) upon oral infection with *P*. *entomophila* (*Pe*) or *Ecc15*. Statistical analysis was performed using a log-rank test. At least 50 flies were used. N.S., not significant. (B) Cell-death was quantified by propidium iodide staining. Results represent the percentage of dead cells (propidium iodide-positive nuclei) in the midguts of flies infected for 4 h with *P*. *entomophila* (*Pe*). Results represent the mean of 10 independent experiments. Statistical analysis was performed by one-way analysis of variance followed by Bonferroni correction for multiple comparisons to evaluate the pairwise difference. UC, unchallenged. (C) A schematic model of the TG-mediated peritrophic matrix formation. TG crosslinks drosocrystallin (Dcy) on the peritrophic matrix (PM). Crosslinked drosocrystallin is not digested by AprA, and the crosslinked drosocrystallin strengthens the peritrophic matrix to function as a physical barrier against exotoxins of pathogenic microbes.

The survival rate of flies ingesting *P*. *entomophila* was analyzed. No significant differences were observed between gut-specific *TG*-knockdown flies and control flies after ingesting a non-lethal pathogen, *Erwinia carotovora carotovora 15* (*Ecc15*), but gut-specific *TG*-knockdown flies had a significantly shorter lifespan than control flies after ingesting *P*. *entomophila*, and the lifespan returned to the control level after ingesting *AprA*-knockout *P*. *entomophila* (Fig 5A). In a previous study, we found that TG-induced dampening of the immune-eliciting signals in the gut and *TG*-RNAi (*NP1*>*TG IR*)-induced shortened lifespan occurred at least 7 days after eclosion [10]. Here, we confirmed that *TG*-RNAi itself did not affect the survival rate in the time span of ~5 days after eclosion (Fig 5A, *TG*-RNAi + sucrose). These data indicate that TG is involved in host defense in the fly gut after infection with *P*. *entomophila* to preserve the proteolytic digestion of drosocrystallin. Protease AprA is a member of the metzincin superfamily and was originally identified in *P*. *aeruginosa* [31,32]. Flies injected with or ingesting *P*. *entomophila* have a shorter lifespan than untreated flies, because AprA facilitates bacterial survival by degrading antimicrobial peptides produced by host immunity [2] and activates monalysin by cleaving the N-terminal pro-peptide [24]. In the present study, we demonstrate for the first time that AprA is directly involved in degrading the peritrophic matrix protein drosocrystallin to shorten the lifespan of *Drosophila*. On the other hand, monalysin causes apoptosis of gut epithelial cells [7,24]. To determine the cause of the shortened lifespan of *TG*-RNAi flies after ingestion of wild-type *P*. *entomophila*, we evaluated gut epithelial cell death. In this experiment, 3 to 5-day old adult flies were used because of the negligible effect of commensal community on the survival rate of *TG*-RNAi flies (Fig 5A). After ingesting *P*. *entomophila*, a significant number of dead cells was detected in the gut, and the ratio of cell death was increased in gut-specific *TG*-knockdown flies (Fig 5B). These findings demonstrate that TG is essential for protection against pathogenic bacterial infection in the gut (Fig 5C).

### Conclusion

Drosocrystallin non-covalently self-associated to form fiber-like structures in the presence of Ca^2+^. Non-crosslinked fibers were not stable and were degraded more quickly by AprA than the crosslinked fibers. TG stabilized the drosocrystallin fibers through intermolecular crosslinking. Such a crosslinking reaction could "mask" potential proteolytic cleavage sites of AprA. Peritrophic matrix proteins, such as insect intestinal mucin and chitin-binding proteins containing multiple chitin-binding domains, are proposed to form a bridge-like structure on chitin fibers on the peritrophic matrix in *T*. *ni* [26,28]. There is no genetic evidence for involvement of these peritrophic matrix proteins with multiple chitin-binding domains in peritrophic matrix formation in *Drosophila*, but the TG-catalyzed crosslinked fibers could promote the formation of a rigid peritrophic matrix structure to protect against exotoxins. Importantly, flies die within 5 h after injection of purified AprA into the hemocoel [2]. Ingestion of a high concentration of AprA has little effect on fly survival, and thus AprA itself is not critical for the virulence of naturally infecting *P*. *entomophila* [2]. These findings suggest that the peritrophic matrix inhibits the penetration of AprA secreted by *P*. *entomophila* from the gut lumen into the ectoperitrophic space. In addition, fluorescein isothiocyanate-labeled dextran-feeding assays performed in our previous study indicated increased permeability of the peritrophic matrix in systemic *TG*-RNAi flies [2]. In the present paper, we did not examine the survival of gut-specific *TG*-knockdown flies after ingesting monalysin and/or AprA, but the TG-mediated crosslinking of drosocrystallin in the peritrophic matrix clearly reduced AprA-mediated peritrophic matrix damage and blocked the movement of monalysin and other virulent factor(s) from the endoperitrophic space into the ectoperitrophic space. The drosocrystallin fibers crosslinked by TG, but not non-crosslinked drosocrystallin fibers, appear to form an important physical barrier against exotoxins of invading pathogenic microbes in the *Drosophila* gut.

## Materials and Methods

### Fly stocks

Flies were maintained on standard yeast medium at 25°C. *Da-GAL4* and *w* ^*1118*^ flies were obtained from the Bloomington Stock Center (Bloomington, IL). *NP1-GAL4* flies were obtained from the *Drosophila* Genetics Resource Center (Kyoto, Japan). *UAS-TG IR* flies were obtained from Dr. Ryu Ueda at the National Institute of Genetics (Mishima, Japan). Strain *w* ^*1118*^ was used as a control strain.

### Bacterial stocks

*P*. *entomophila* L48 [23], *P*. *entomophila* ^*ΔaprA*^ [2], and *Ecc 15* were grown in Luria-Bertani (LB) medium for all experiments. Bacteria were grown at 29°C and allowed to reach the stationary phase. Cells were then concentrated at OD_600_ = 200 except when indicated.

### Infection and survival assays

For oral infection, female flies were starved for 2 h at 29°C. *Ecc 15* or *P*. *entomophila* (OD_600_ = 200) was added to a filter disk (Whatman) that completely covered the surface of the standard fly medium, and the flies were placed on the medium. Flies were maintained at 29°C, and mortality was monitored at different time-points.

### Expression of recombinant drosocrystallin

To construct expression vectors, cDNA fragments were amplified by polymerase chain reaction (PCR). An amplimer encoding the *drosocrystallin*-coding sequence without a putative signal sequence (1–60) was inserted into expression vector pET-22b (Novagen) between the *Nde*I and *EcoR*I sites. The construct was verified by DNA sequencing. The construct, which contained C-terminal His-tags, was expressed in the *E*. *coli* strain BL21 (DE3) (Novagen). Bacteria were cultured in LB medium, and expression was induced by the addition of isopropyl-β-D-thiogalactoside at a final concentration of 1 mM at 15°C for 24 h. Bacterial pellets were harvested by centrifugation and sonicated in 10 ml of 20 mM Tris-HCl, pH 8.0, 200 mM NaCl containing 1% Nonidet P-40 and 1 mM phenylmethylsulfonylfluoride. After sonication, the supernatants were recovered by centrifugation and purified according to the manufacturer’s protocol using Ni-NTA agarose (Qiagen). To produce protein insensitive to TG, all lysine residues of drosocrystallin were substituted with arginine by PCR-based site-directed mutagenesis. Each amino acid substitution was generated by PCR using specific 5’-phosphorylated primers. The lysine-substituted mutation was verified by DNA sequencing and expressed in BL21 (DE3)/pLysS by the same method as used for the wild type. K35R-sense primer, GGTCCTCCAACCTTCAGCAG; K35R-antisense primer, TGGCTAGCTGGTTAAGATCG; K92, 96, 103R-sense primer, GGCGGCAGGAGGAGAGGCGCGATGGCGACCTGGTCAGGGGT; K92, 96, 103R-antisense primer, TGTCATCGCCAGTCAGCGAG; K135R-sense primer, GGCAGCGTCTGGATGAGCAG; K135R-antisense primer, TAGACACAATGGCATTGAAG; K470R-sense primer, GGAACTGGCCGAATTCGAGCTCCGTCGACAGGCTT; K470R-antisense primer, TAGAGCGACGTTCGGCACTG.

### Expression of recombinant TG

The whole sequence encoding the *TG* gene was cloned into expression vector pET-22b. The construct was verified by DNA sequencing. The construct, which contained no tags, was expressed in the *E*. *coli* strain BL21 (DE3). Bacteria were cultured in LB medium, and expression was induced by the addition of isopropyl-β-D-thiogalactoside at a final concentration of 30 μM at 15°C for 24 h. Bacterial pellets were harvested by centrifugation and sonicated in 50 mM Tris-HCl, pH 8.8, 50 mM NaCl, 10 mM dithiothreitol (DTT), 2 mM EDTA, 10% glycerol, and 1% 3-[(3-cholamidepropyl)dimethylammonio]-1-propanesulphonate. After sonication, the supernatants were recovered by centrifugation. Then, buffer was exchanged with 50 mM Tris-HCl, pH 8.0, 10 mM DTT, 1 mM tris(2-carboxyethyl)phosphine, and 0.5 mM EDTA using a Sephadex G-25 Superfine column, and stored at -80°C before use.

### Expression of wild-type monalysin

The whole sequence encoding the *monalysin* gene was generated by PCR with C-terminal HAT encoding primers and cloned into expression vector pET-15b. The construct was verified by DNA sequencing. The construct was expressed in the *E*. *coli* strain Rosseta-gami B. Bacteria were cultured in LB medium, and expression was induced by the addition of isopropyl-β-D-thiogalactoside at a final concentration of 0.1 mM at 18°C for 24 h. Bacterial pellets were harvested by centrifugation and sonication buffer (50 mM Tris-HCl, pH 8.0, 500 mM NaCl, 1 mM phenylmethylsulfonylfluoride, 0.5 mM lysozyme) was added and frozen. After thawing and sonicating the pellets, the supernatant was recovered by centrifugation. The supernatant was purified using HisTrap crude FF column (1 mL, GE Healthcare). After purification, the buffer was exchanged with PBS using Sephadex G-25 Superfine column.

### Preparation of a polyclonal antibody against drosocrystallin

To prepare the polyclonal antibody, recombinant drosocrystallin without a putative signal sequence (61–472) was expressed in *E*. *coli* strain BL21 (DE3) (Novagen). An inclusion body containing the recombinant protein was isolated and subjected to SDS-PAGE under reducing conditions, and negatively stained. The protein band corresponding to the recombinant protein was excised from the gel band recovered by electroelution for immunization of rabbits (MBL International). The polyclonal antibody was purified sequentially from the anti-serum using Protein A Sepharose CL-4B (GE Healthcare) and antigen-conjugated Affi-Gel 15 (Bio-Rad Laboratories).

### Chitin-binding assay

The recombinant proteins were mixed with chitin in 50 mM Tris-HCl, pH 7.5, and 150 mM NaCl, and incubated at 4°C for 30 min. Supernatants were separated by centrifugation and precipitates were washed with the same buffer. Proteins bound to chitin were eluted with 10% acetic acid. Eluted fractions (100 μL each) were evaporated using a speed-vac (Labconco). Input, bound, and unbound fractions were subjected to SDS-PAGE and detected by Coomassie brilliant blue staining. The relative intensity of each fraction compared to the input protein was calculated by ImageJ software.

### Incorporation of biotin pentylamine into wild-type drosocrystallin

Recombinant proteins were incubated with TG in 50 mM Tris-HCl, pH 8.5, containing 10 mM CaCl_2_, 10 mM DTT, and 500 μM biotin pentylamine at 37°C for 1 h. Following the reaction, the aliquots were subjected to SDS-PAGE and electroblotted on a polyvinylidene difluoride membrane. After blocking with 20 mM Tris-HCl, pH 7.5, 150 mM NaCl, and 5% dry milk, the membrane was incubated at room temperature for 1 h with the horseradish peroxidase-conjugated streptavidin diluted 1:1,000 with blocking buffer, followed by development with Chemi-Lumi One-Super reagent (Nacalai Tesque).

### Quantification of MDC incorporation

Recombinant proteins were incubated with TG in 50 mM Tris-HCl, pH 8.5, 10 mM CaCl_2_, 10 mM DTT, and 5 mM MDC at 37°C for the durations indicated in Fig 1B. Following the reaction, aliquots were subjected to SDS-PAGE and visualized by ultraviolet irradiation. Band intensity was calculated using ImageJ software.

### SDS-PAGE and western blotting

SDS-PAGE was performed in slab gels according to the method of Laemmli. Precision Plus protein standards (Bio-Rad Laboratories) were used to determine the apparent molecular masses. Protein bands were visualized by Coomassie brilliant blue staining. Samples were subjected to SDS-PAGE and transferred to a polyvinylidene difluoride membrane. After blocking with 5% dry milk, the membrane was incubated at room temperature for 1 h with the anti-drosocrystallin antibody and then with the secondary antibody (horseradish peroxidase-conjugated goat anti-rabbit IgG; Bio-Rad Laboratories), followed by development with Chemi-Lumi One, Chemi-Lumi One-super (Nacalai), or WesternBright Sirius (Advansta). For detection of the His-tag, horseradish peroxidase-conjugated anti-6 × His tag antibody (MBL International) was used. Chemifluorescence was detected using an Omega Lum G fluorescence imager (Aplegen) or X-ray film.

### Detection of crosslinked drosocrystallin

Wild-type drosocrystallin or the KR mutant was incubated with TG in 50 mM Tris-HCl, pH 8.5, 10 mM CaCl_2_, and 10 mM DTT at 37°C for 1 h. Following the reaction, samples were subjected to SDS-PAGE and detected by Western blotting using anti- 6 × His tag antibody. Guts from wild-type and *TG-*knockdown flies (*Da>TG IR*) were homogenized in 50 mM Tris-acetate, pH 7.5, 1% Nonidet P-40, and protein inhibitor cocktail (Nacalai Tesque), and centrifuged at 15,000 rpm at 4°C for 15 min to collect the supernatant. The supernatant was precipitated by 10% trichloroacetic acid, subjected to SDS-PAGE, and detected by Western blotting using anti-drosocrystallin antibody.

### Live imaging

Quantification of dead cells was performed as follows: 4 h after ingestion of *P*. *entomophila*, the guts were dissected and stained with Hoechst 33342 (1:1,000, Dojindo Molecular Technologies) and propidium iodide (1:2,000, Life Technologies). Pictures were obtained with a fluorescence microscope. From these pictures, 100 Hoechst 33342-stained nuclei, representing all nuclei, were randomly defined and the number of propidium iodide-positive nuclei, representing dead cells, was determined. Three parcels per gut were analyzed. Results represent the mean of 10 independent experiments. In this experiment, 3 to 5-day-old adult flies were used.

### Preparation of AprA

Culture supernatant from the wild-type *P*. *entomophila* was fractionated using ÄKTA start with a HiPrep 16/60 Sephacryl S-100 HR column (GE Healthcare). Each fraction was incubated with the wild-type recombinant. The sample from fraction No. 26 was dialyzed with 20 mM Tris-HCl, pH 7.5, and applied to a DEAE Sepharose CL-6B column (1×2 cm). The flow-through fraction was applied to a CM Sepharose CL-6B column (1×2 cm). After washing with 20 mM Tris-HCl, pH 7.5, the protein was eluted with a linear NaCl gradient (100–500 mM) in the same buffer.

### Observation of drosocrystallin fibers

One microgram of wild-type drosocrystallin in 50 mM Tris-HCl, pH 8.5, and 10 mM DTT with 10 mM CaCl_2_ or 50 mM EDTA was placed on a coverslip and incubated at 37°C for 1 h with or without TG. Next, the culture supernatant from *P*. *entomophila* and/or HAT-tagged monalysin was added to the coverslip and incubated at 37°C for 1 h. After incubation, the proteins were fixed with 4% paraformaldehyde for 20 min, washed with 20 mM Tris-HCl, pH 7.5, 150 mM NaCl, and blocked with 2% bovine serum albumin in the same buffer. The proteins were then incubated for 1 h with anti-His tag monoclonal antibody (MBL International) for wild-type drosocrystallin and the anti-HAT polyclonal antibodies (GenScript) for monalysin. For detection, CF488 or CF568-conjugated goat anti-mouse secondary antibody (Biotium) and CF568-conjugated goat anti-rabbit secondary antibody (Biotium) were used. The proteins were imaged with a ZOE fluorescence microscope (Bio-Rad) for detection of the structure of wild-type drosocrystallin or MZ10 F (Leica) for calculating the mean gray value. The mean gray value of the signal for wild-type drosocrystallin was calculated using ImageJ software. The sum of gray values in the protein-coated area was divided by the number of pixels. The mean gray value of uncoated-area was subtracted from the value of the protein-coated area.
