# Correction: Crosslinking of a Peritrophic Matrix Protein Protects Gut Epithelia from Bacterial Exotoxins

**DOI:** 10.1371/journal.ppat.1005670

**Published:** 2016-06-01

**Authors:** Toshio Shibata, Kouki Maki, Jinki Hadano, Takumi Fujikawa, Kazuki Kitazaki, Takumi Koshiba, Shun-ichiro Kawabata

In Fig 3E, the labels on the graph in the lower panel are swapped. The solid line should be labeled Pe sup and the dashed line should be labeled Pe^ΔaprA^ sup. Please see the corrected Fig 3 here.

**Fig 3:**
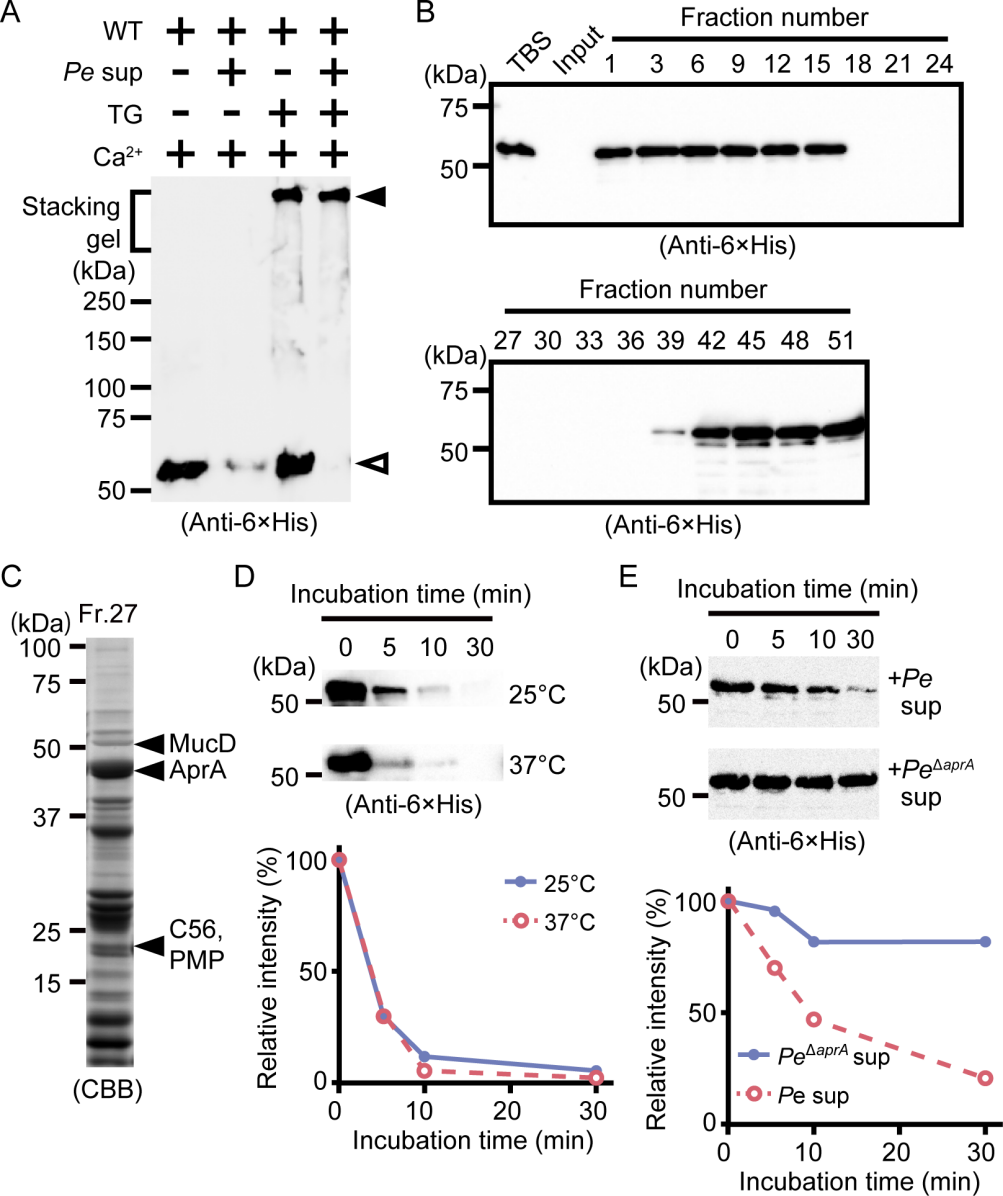
Polymerized drosocrystallin protects against AprA. (A) Wild-type drosocrystallin (WT) was incubated at 37°C for 30 min with or without TG, and then the culture supernatant from P. entomophila (Pe) was added, and the mixture was subjected to SDS-PAGE in 10% TGX FastCast gels. Open arrowhead, the monomeric recombinant; closed arrowhead, the crosslinked recombinant. Data are representative of at least three independent experiments. (B) Wild-type drosocrystallin was subjected to SDS-PAGE in 10% slab gels and detected by Western blotting after incubating with each fraction obtained by gel filtration of the culture supernatant from P. entomophila. (C) Fraction No. 27 from the gel filtration was subjected to SDS-PAGE in 15% slab gels, and proteases in this fraction were identified by liquid chromatography tandem mass spectroscopy analysis. (D) Wild-type drosocrystallin was incubated with purified AprA at 25°C or 37°C and analyzed by SDS-PAGE in 10% slab gels, and detected by Western blotting using anti-6 × His tag antibody (upper panel). Western blotting data are representative of four independent experiments. The relative intensity of each band compared to that of the untreated protein (0 min) was calculated using ImageJ software (lower panel). (E) Wild-type drosocrystallin was incubated with the culture supernatant from P. entomophila (Pe) or the AprA-knockout strain (PeΔaprA), analyzed by SDS-PAGE in 10% slab gels, and detected by Western blotting using anti-6 × His tag antibody. Western blotting data are representative of three independent experiments (upper panel). The relative intensity of each band compared to that of the untreated protein (0 min) was calculated using ImageJ software (lower panel).
